# Anti-Scarring Properties of Different Tryptophan Derivatives

**DOI:** 10.1371/journal.pone.0091955

**Published:** 2014-03-17

**Authors:** Malihe-Sadat Poormasjedi-Meibod, Ryan Hartwell, Ruhangiz Taghi Kilani, Aziz Ghahary

**Affiliations:** Division of plastic surgery, Department of Surgery, University of British Columbia, Vancouver, British Columbia, Canada; Helmholtz Zentrum München/Ludwig-Maximilians-University Munich, Germany

## Abstract

Hypertrophic scars are associated with prolonged extracellular matrix (ECM) production, aberrant ECM degradation and high tissue cellularity. Routinely used antifibrotic strategies aim to reduce ECM deposition and enhance matrix remodeling. Our previous study investigating the antifibrotic effects of indoleamine2, 3 dioxygenase (IDO) led to the identification of kynurenine (Kyn) as an antiscarring agent. A topical antifibrogenic therapy using Kyn is very attractive; however, it is well established that Kyn passes the blood brain barrier (BBB) which causes complications including excitatory neuronal death. Here we investigated the antiscarring properties of kynurenic acid (KynA), a downstream end product of Kyn that is unlikely to pass the BBB, as an effective and safe replacement for Kyn. Our results indicated that while not having any adverse effect on dermal cell viability, KynA significantly increases the expression of matrix metalloproteinases (MMP1 and MMP3) and suppresses the production of type-I collagen and fibronectin by fibroblasts. Topical application of cream containing KynA in fibrotic rabbit ear significantly decreased scar elevation index (1.13±0.13 vs. 1.61±0.12) and tissue cellularity (221.38±21.7 vs. 314.56±8.66 cells/hpf) in KynA treated wounds compared to controls. KynA treated wounds exhibited lower levels of collagen deposition which is accompanied with a significant decrease in type-I collagen and fibronectin expression, as well as an increase in MMP1 expression compared to untreated wounds or wounds treated with cream only. The results of this study provided evidence for the first time that KynA is promising candidate antifibrogenic agent to improve healing outcome in patients at risk of hypertrophic scarring.

## Introduction

Wound healing in skin is a highly regulated process the outcome of which is defined by a fine balance between ECM deposition and degradation. Abnormal matrix deposition and remodeling, leading to ECM accumulation at the wound site, play a pivotal role in hypertrophic scar (HSC) formation [1], [2]. A study by Ghahary *et al*. [3] revealed higher levels of collagen-I, collagen-III and fibronectin expression in HSC tissue compared to normal skin. They also showed that collagenase mRNA expression and activity in the fibroblast conditioned medium is significantly reduced in HSC fibroblasts compared to the normal ones [4]. Recent studies [5], [6], [7] demonstrate that localized application of agents that are targeted to suppress matrix accumulation provide an approach to specifically reduce scarring.

Indoleamine2, 3 dioxygenase (IDO), a cytosolic enzyme which catalyses the first and rate-limiting step in the tryptophan catabolism to N-formylkynurenine, is widely known for its immuno-regulatory properties [8], [9], [10]. A previous study by our group revealed that local IDO expression not only protects the transplanted xenogeneic skin substitute from immune rejection but also reduces scarring in a rabbit ear model [11]. Further studies by our group revealed that the antiscarring properties of IDO, which are dependent on Kyn accumulation, are in part mediated by increasing the expression of MMPs by dermal fibroblasts. This initial result that kynurenine could reduce dermal fibrosis was further confirmed with topical application of a kynurenine cream that again improved healing quality in a fibrotic animal model [12].

Although Kyn demonstrated efficacy in preventing dermal fibrosis, possible adverse effects may result if higher than normal levels are achieved systemically as a therapeutic. Circulatory Kyn is actively transported across the BBB by the large neutral amino acid carriers [13]. Kyn and tryptophan compete for the same carrier to be transported via the BBB; as such, elevated levels of Kyn in circulation may lead to a significant depletion in the tryptophan pool of the brain. Tryptophan deficiency is associated with dysregulation of serotonin (5-hydroxytryptamine) metabolism which contributes to many psychiatric disorders [14], [15]. The transported Kyn is mainly metabolized to 3OH-kynurenine, 3OH-anthranilic acid and quinolinic acid in the brain [16]. These metabolites either directly cause apoptotic or necrotic neuronal death [17], [18], [19], or induce excitotoxic neuronal death via binding to N-methyl-D-aspartate (NMDA) receptors in the brain [20], [21]. The potential adverse effects of Kyn prompted our search for an antifibrogenic compound with less potential risks. Contrary to other Kyn metabolites KynA, which poorly crosses the BBB [13], has neuroprotective properties and reduces the excitotoxin-induced neuronal death by antagonising the ionotropic glutamate receptors [19] and the α7 nicotinic receptors [22]. As a potentially fitting candidate replacement for Kyn the purpose of this study was to evaluate the antifibrotic properties of KynA both *in vitro* and in a fibrotic rabbit ear model. Given that molecular toxicity can, in many ways, be stereospecific we also evaluated the effect of L- and D-isomers of Kyn (L-Kyn and D-Kyn, respectively) on ECM expression in order to find the active enantiomer of the drug. Application of one active enantiomer only instead of the racemic form of the drug provides the possibility to reduce the administered dosage and clinically significant side-effects while having the desired biological effect [23], [24]. The results of this study provide evidence for the first time that KynA is a potent antifibrogenic replacement for Kyn both *in vitro* and in a fibrotic animal model.

## Materials and Methods

### Ethics statement

All methods and procedures, as well as the use of animals and tissue specimens derived from animals and humans, whether or not obtained solely for the purpose of this study are approved by both Human and Animal Ethics Committees of the University of British Columbia. Written consent from informed donors was received prior to conducting any sampling of tissue specimens.

### Cell culture

Foreskin samples were obtained from healthy patients undergoing elective circumcision. Human primary keratinocytes and fibroblasts were harvested from foreskin samples as described previously [25]. Fibroblasts were cultured in Dulbecco's Modified Eagle's Medium (DMEM, GIBCO, Grand Island, NY) with 10% fetal bovine serum (FBS). Keratinocytes were cultured in Keratinocyte serum-free medium (KSFM, Invitrogen Life Technologies, Carlsbad, CA) supplement with bovine pituitary extract (25 ng/ml, BPE) and epidermal growth factor (0.2 ng/ml, EGF, GIBCO). Keratinocytes and fibroblasts at passage 4–7 were used in all experiments in this study.

### RNA extraction and Quantitative real time PCR (Q-PCR)

In order to determine the effect of kynurenines on ECM component gene expression, dermal fibroblasts were cultured and treated with increasing concentrations (6.25, 12.5, 25, 50, 100 and 150 μg/ml) of KynA, Kyn, L-Kyn or D-Kyn for 24 hours. Total RNA was isolated using the GeneJET RNA Purification Kit (Fermentas International Inc, Thermo Fisher Scientific, Ottawa, ON) according to the manufacturer's instructions. Total RNA (5 μg) was reverse transcribed into cDNA using a Superscript II First Strand cDNA Synthesis kit (Invitrogen). Q-PCR was carried out on the Applied Biosystems 7500 Fast Real-Time PCR System, using the SYBR Green PCR Master-Mix kit (Applied Biosystems, Warrington, UK). The following cycling conditions were used for Q-PCR: 95°C/15 min with 40 cycles of 95°C/1 min, 55°C/30 sec, and 72°C/30 sec. The following primers were used for Q-PCR reactions: MMP1 5′-CTCAGGATGACATTGATGGC-3′ and 5′-CCCCGAATCGTAGTTATAGC-3′, MMP3 5′- TGGCATTCAGTCCCTCTATRGG-3′ and 5′-AGGACAAAGCAGGATCACAGTT-3′, MMP3 5′-TTCCGCCTGTCTCAAGATGATAT-3′ and 5′-AAAGGACAAAGCAGGATCACAGTT-3′, Col-1α1 5′-CTGGAATGAAGGGACACA-3′ and 5′-CCATTGGCACCTTTAGCA-3′, and fibronectin 5′-GATAAATCAACAGTGGGAGC-3′ and 5′-CCCAGATCATGGAGTCTTTA-3′. Glyceraldehyde-3-phosphate dehydrogenase (GAPDH) was used as the reference gene. GAPDH 5′-GACAAGCTTCCCGTTCTCAG-3′ and 5′-CAATGACCCCTTCATTGACC-3′ (Invitrogen).

### Preparation of cell lysates and western blotting

Dermal fibroblasts were cultured and treated as described above for 48 hours. The whole cell proteins were separated by running the samples on 10% SDS-polyacrylamide gel and then transferred to polyvinylidene difluoride (PVDF) membrane (Millipore, Bedford, MA). Membranes were blocked and probed for MMP1, type-I collagen and fibronectin using rabbit-anti MMP1 (1∶2000 dilution, Abcam, Cambridge, MA, USA), mouse-anti collagen-I (1∶100, Developmental Studies Hybridoma Bank), and rabbit-anti fibronectin (1∶1000, Santa Cruz Biotechnology, CA). Horseradish peroxidase (HRP)-conjugated goat anti-rabbit Ab (1∶3000 dilution, Bio-Rad) and HRP-conjugated goat anti-mouse Ab (1∶3000 dilution, Bio-Rad) were used as the secondary antibodies. β-actin was used as the loading control.

Conditioned medium (30 μl) from the untreated and treated dermal fibroblasts were subjected to Western blotting for detection of secreted MMP3 protein. Mouse-anti MMP3 antibody (1∶2000, R&D Systems, Minneapolis, MN) and HRP-conjugated goat anti-mouse Ab were used as the primary and secondary antibodies, respectively.

### MMP activity assay

The MMP activity in the conditioned medium of control and treated fibroblast was determined using the SensoLyte Plus 520 generic MMP Assay Kit (Anaspec) according to the manufacturer's instructions following 48 hours of cell treatment with 100 μg/ml of either KynA, Kyn, L-Kyn or D-Kyn. The kit is designed to detect the activity of a variety of MMPs, including MMP-1, 2, 3, 7, 8, 9, 12, 13, and 14.

### Collagen Accumulation Assay by Sirius Red staining

To evaluate the effect of different tryptophan derivatives on soluble collagen level in the fibroblast conditioned medium cells were treated with increasing concentrations (50 and 150 μg/ml) of Kyn or KynA. Following 96 hours of incubation Sirius Red collagen detection kit (Chondrex, Inc.) was used to measure the amount of collagen in the cell culture medium, following the manufacturer's instructions. Cells were harvested and counted after 96 hours of incubation. The amount of soluble collagen was normalized based on the cell number.

### Lasting effect study

To determine the effect of a single treatment Kyn and KynA on MMP expression over time, fibroblasts were cultured and treated with 100 μg/ml of KynA or Kyn for 48 hours. Cells were washed with Phosphate buffered saline (PBS) and cultured in fresh medium without treatment. Cells were harvested immediately, 12, 24, and 48 hours after treatment removal and the presence of MMP1 was evaluated in cell lysate by Western blotting.

### Cell proliferation assay

To evaluate the effect of different tryptophan derivatives on dermal cell proliferation rate, fibroblasts and keratinocytes were seeded on 6-well plates (30×10^3^ and 20×10^3^ cells/well, respectively) and treated with KynA or Kyn (100 μg/ml). Cells were harvested and total number of the cells was counted after 36, 72 and 108 hours of treatment.

### Live/dead, viability/cytotoxicity assay

Cells were plated in 6-well plates (250×10^3^ cells/well), cultured and treated with increasing concentrations (50 and 150 μg/ml) of Kyn or KynA. After 3 days of incubation cell viability was evaluated by flow cytometry using the Live/Dead, Viability/Cytotoxicity assay kit for mammalian cells (Invitrogen). In this assay ethidium homodimer (EthD-1), a red fluorescent nucleic acid dye, stains dead cells and those undergo apoptosis; while, clacein AM is converted to a green fluorescent compound by active intracellular esterase in live cells. Percentage of dead cells plus cells with damaged (porous) cellular membrane was quantitatively evaluated based on the protocol suggested by the manufacturer (Molecular Probes, Invitrogen, Mississauga Canada).

### *In vitro* wound healing scratch assay

Fibroblasts and keratinocytes were seeded on 12-well plates. At 95% confluency a scratch wound was made across each well with a 200-μl pipette tip and the cells were washed with PBS. The cells were incubated in fresh medium containing either KynA or Kyn (100 μg/ml). Photographs were taken immediately, 12, and 24 hours after treatment and the total number of cells migrated into the open wound area was counted manually. Cell migration in treated samples was evaluated by quantifying the total number of cells migrated to the open wound area and compared with those of control.

### Hypertrophic scar animal model and treatments

The fibrotic rabbit ear model established by Moriss *et al*. [26] was used as the cutaneous scarring model. Briefly, 4 female New Zealand white rabbits were anesthetized and prepared for wounding. Four full thickness wounds (8 mm) were created down to the bare cartilage on the ventral surface of each ear. The wounds were covered for three days using a Tegaderm dressing (3 M, St. Paul, MN). Treatments were started following the initiation of granulation tissue formation. Wounds were either left untreated to heal by secondary intention, treated with cream alone or cream containing either KynA or Kyn (500 μg/ml). Treatments were applied once a day for 35 consecutive days. Animals were sacrificed on day 35 post-wounding and tissue was harvested for histology.

### Tissue processing and determination of scar elevation index (SEI) and epidermal thickness index (ETI)

Scars were harvested with a 0.5 cm margin of surrounding unwounded tissue and bisected through the maximum point of scar hypertrophy on visualization and palpation. Half of the samples were fixed in 10% buffered formalin solution, and embedded in paraffin. Sections (5 μm) were rehydrated and subjected to Haematoxylin-Eosin (H&E) staining.

The SEI, ratio of the scar's newly formed dermal area to the area of unwounded dermis, was represents the degree of dermal hypertrophy of each scar. An SEI value greater than 1.0 depicts a raised or hypertrophic dermis. The ETI was used to express the degree of scar epidermal hypertrophy. The entire cross section of the scar, which corresponded to a total of five fields (400X), was measured for epidermal thickness. The five fields of uninjured skin from both sides of the scar were also evaluated to determine the epidermal thickness in normal skin. The ratio between the averaged epidermal height in scar tissue and the averaged epidermal height in the normal uninjured skin was measured to determine the ETI. The ETI value greater than 1.0 depicts epidermis hypertrophy.

### Collagen staining and tissue cellularity

In order to determine the amount of collagen deposition, paraffin embedded sections were stained for collagen with Masson's Trichrome as described previously [6]. Keratin and muscle fibers are stained red, collagen fibers are stained blue and cell cytoplasm and nuclei are stained light pink and dark brown, respectively. To evaluate the effect of topical KynA and Kyn on dermal cellularity, five randomly chosen fields of dermis were photographed under 200X magnification. Photos were subsequently coded and randomized, overall dermal cellularity was quantified by manually counting the number of nuclei per high-power field (hpf) by a blinded observer. The counts from the five fields were averaged and used for comparisons between wounds (n = 4 for each treatment or control group).

### MMP-1, type-I collagen and fibronectin expression in wounds

Total RNA was extracted from half of the scar tissue using Trizol reagent according to the manufacturer's instructions (Invitrogen). Following DNase treatment and cDNA synthesis expression of MMP1, type-I collagen and fibronectin were evaluated in samples using Q-PCR as described above. The following primers were used for Q-PCR reactions: MMP1 5′-TCTGGCCACATCTGCCTAATGG-3′ and 5′-AGGGAAGCCAAAGGAGCTGTG-3′, Col-1α1 5′-TGTTCAGCTTTGTGGACCTCC-3′ and 5′-TTCGCCTTCACTGTACCGGAC-3′, and fibronectin 5′-AGCAGCTTTGTGGTCTCGTGG-3′ and 5′-TTCGGCCAGGAAGCAAGTCTG-3′ (Invitrogen). GAPDH was used as the reference gene.

### Statistical analysis

Data were expressed as mean±SEM of three or more independent observations. Statistical significance was calculated using a two-tailed unpaired student t-test or a one-way analysis of variance with post hoc test in case of multiple comparisons. P-values<0.05 were considered statistically significant in this study.

## Results

The effect of tryptophan derivatives on the expression of different ECM components (type-I collagen and fibronectin) and key ECM degrading enzymes (MMP1 and MMP3) was evaluated using Q-PCR and Western blotting. Cells treated with increasing doses (6.25, 12.5, 25, 50, 100 and 150 μg/ml) of KynA, Kyn, L-Kyn or D-Kyn were harvested after either 24 or 48 hours for mRNA and protein analysis, respectively.

**Figure 1:**
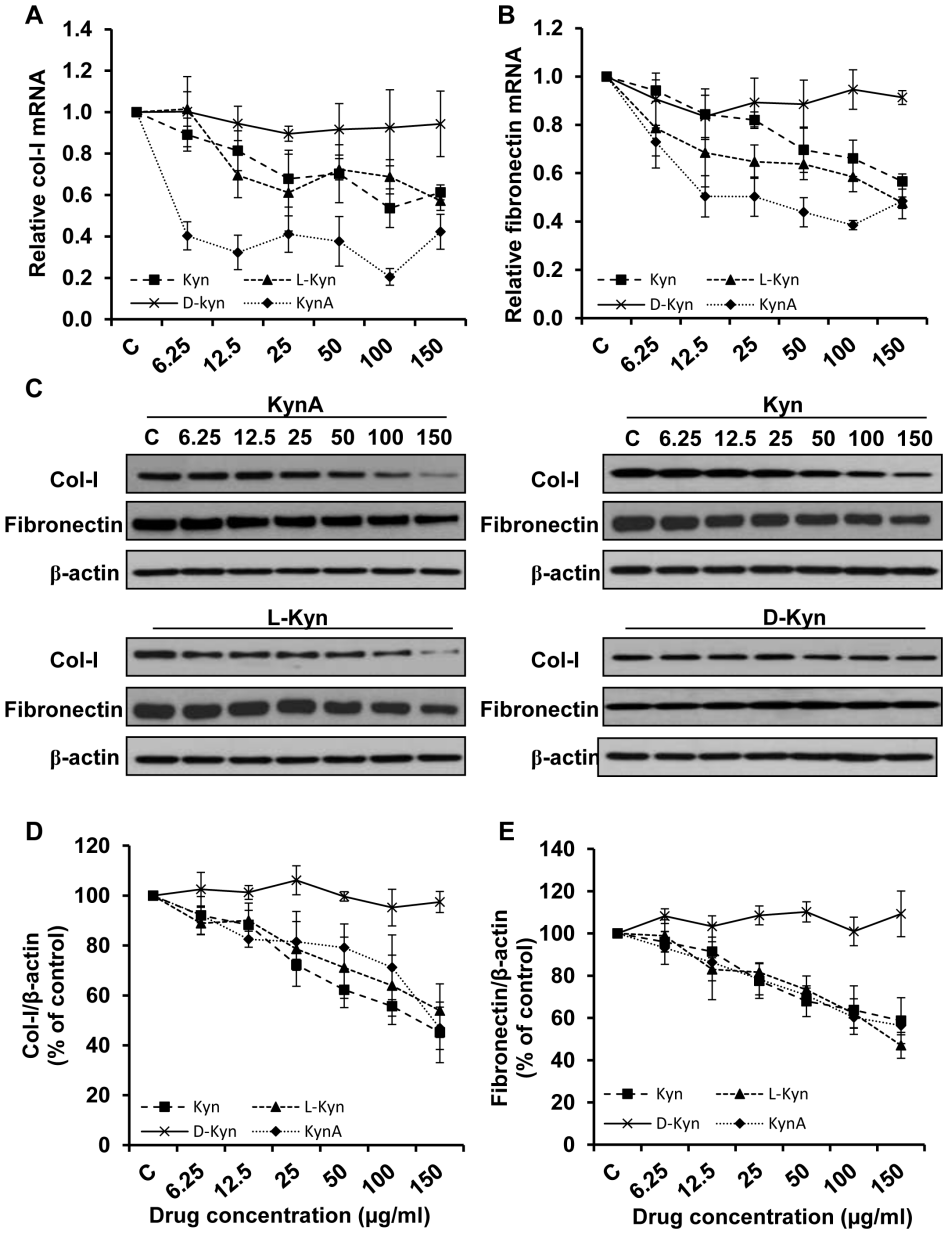
Inhibition of type-I collagen and fibronectin expression in dermal fibroblasts by kynurenines. Type-I Collagen and fibronectin expression at the mRNA and protein level in cultured fibroblasts treated with increasing concentrations (6.25, 12.5, 25, 50, 100, and 150 μg/ml) of KynA, Kyn, L-Kyn or D-Kyn. **A & B**: Relative type-I collagen and fibronectin mRNA expression in treated fibroblasts, respectively. GAPDH was used as the reference gene. **C**: Evaluation of type-I collagen and fibronectin expression at the protein level using Western blotting. **D & E**: The Mean±SEM ratio of type-I collagen and fibronectin density to β-actin at the protein level, respectively. β-actin was used as protein loading control.

The result shown in figure 1A and 1B indicates a dose dependent decrease in type-I collagen and fibronectin mRNA expression respectively in fibroblasts treated with increasing concentrations of KynA, Kyn or L-Kyn relative to that of untreated control. Fibroblast treatment with KynA resulted in a significant decrease in type-I collagen and fibronectin mRNA expression at concentrations as low as 6.25 μg/ml and this reduction remained significant up to 150 μg/ml. Kyn and L-Kyn with concentrations over 25 μg/ml significantly decreased the type-I collagen and fibronectin mRNA expression by fibroblasts. As it is shown in this figure, KynA has the most profound inhibitory effect on fibronectin and type-I collagen mRNA expression among the tested kynurenines. The protein expression analysis similarly showed that KynA, Kyn and L-Kyn suppresses the production of type-I collagen and fibronectin in a concentration dependent fashion (Fig. 1C). Figures 1D and 1E depict the quantitative analysis of the data shown in figures 1C for type-I collagen and fibronectin protein expression, respectively. The difference between control and either KynA, Kyn or L-Kyn treated fibroblast in fibronectin or type-I collagen expression was significant as low as 25 μg/ml and remained significant up to 150 μg/ml tested. KynA, Kyn and L-Kyn demonstrate a comparable suppressive effect on type-I collagen and fibronectin protein expression. Moreover, the presented data generated by Q-PCR and Western blotting revealed that D-Kyn does not have any significant inhibitory effect on type-I collagen or fibronectin expression at the mRNA or protein level.

The effect of KynA and Kyn on the soluble collagen level was evaluated using Sirius Red collagen detection kit. Sirius red dye which binds to the [Gly-X-Y]_n_ helical structure on fibrillar collagen can detect collagen type I, II, III and IV in cell conditioned medium. Consistent with previous data generated by Q-PCR and Western blot analysis, KynA and Kyn significantly reduce the production of soluble collagen in treated fibroblasts compared to the control in a dose depended manner (Fig. S1).

**Figure 2:**
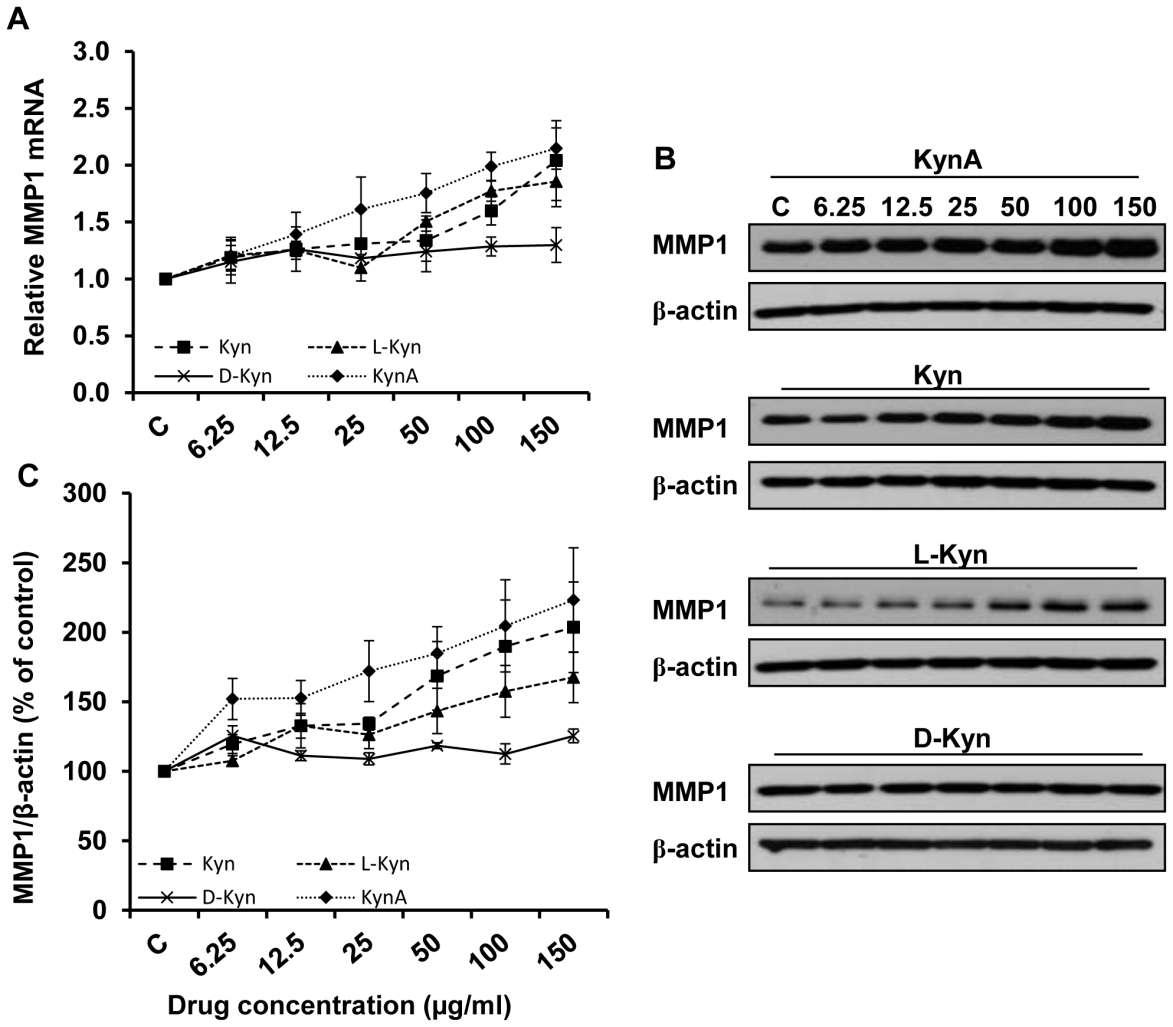
Stimulatory effect of kynurenines on MMP1 expression. **A**: Dermal fibroblasts were treated with increasing doses (6.25, 12.5, 25, 50, 100, and 150 μg/ml) of KynA, Kyn, L-Kyn or D-Kyn. Following 24 hours of treatment cells were collected, and MMP1 expression was determined by Q-PCR after RNA extraction and cDNA synthesis. **B**: Evaluation of MMP1 expression at the protein level by Western blotting after 48 hours of treatment. **C**: The Mean±SEM ratio of MMP1 to β-actin density at the protein level. β-actin and GAPDH were used as loading controls for western blotting and Q-PCR, respectively.

The results obtained from the Q-PCR analysis and Western blotting demonstrate that while KynA, Kyn or L-Kyn treatment increases the MMP1 mRNA and protein levels in a dose depended manner, D-Kyn does not have any noticeable stimulatory effect on MMP1 expression by dermal fibroblasts (Fig. 2A and 2B, respectively). Figure 2C depicts the quantitative analysis of the data shown in figures 2B for MMP1 protein expression. KynA has the highest stimulatory effect on the MMP1 expression as compared to other tryptophan metabolites examined. Also consistent with Q-PCR results, D-Kyn fails to significantly stimulate MMP1 expression in dermal fibroblasts.

**Figure 3:**
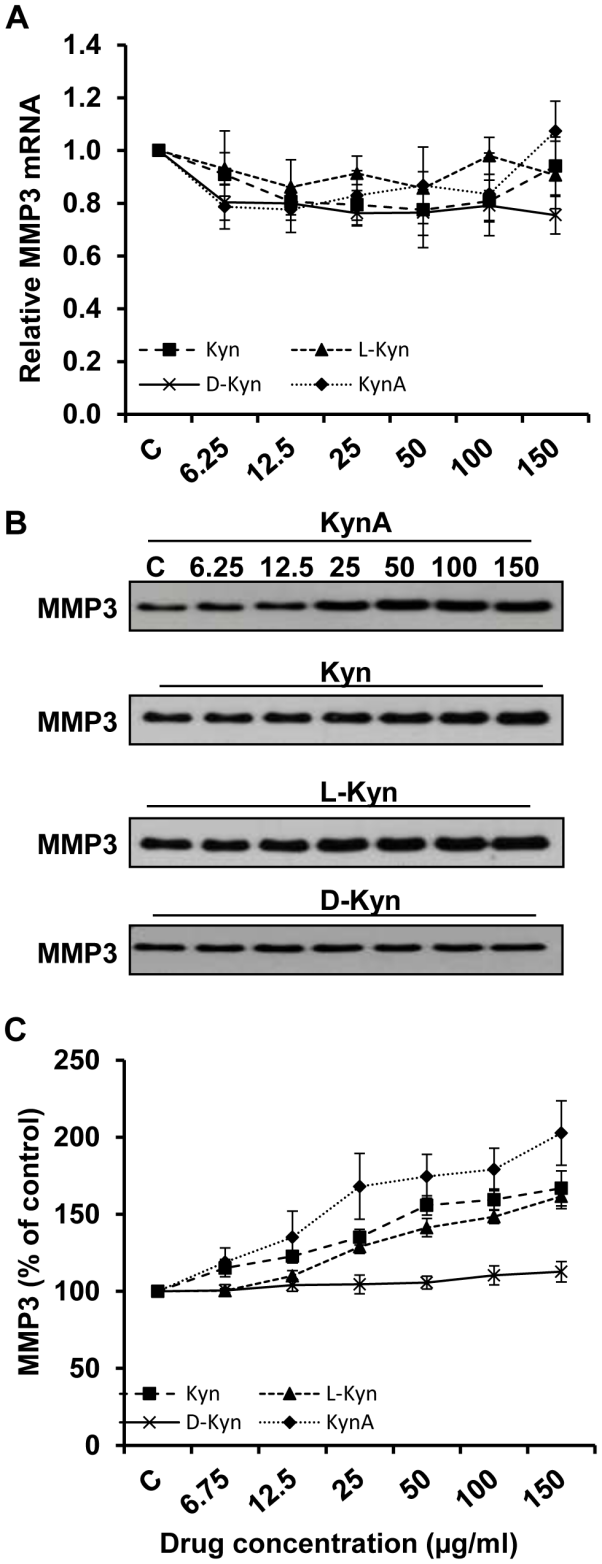
Stimulatory effect of kynurenines on MMP3 secretion by fibroblasts. **A**: Evaluation of MMP3 mRNA expression in fibroblasts treated with increasing doses (6.25, 12.5, 25, 50, 100, and 150 μg/ml) of KynA, Kyn, L-Kyn or D-Kyn following 24 hours of treatment. GAPDH was used as loading control for Q-PCR. **B**: Evaluation of MMP3 presence in the fibroblast conditioned medium using Western blotting after 48 hours of treatment. **C**: The Mean±SEM ratio of MMP3 density at the protein level.

Notably, in contrary to the results of MMP1 mRNA and protein expression, the level of MMP3 mRNA remained relatively unchanged (Fig 3A). The obtained Q-PCR data for MMP3 mRNA expression was confirmed using a second set of primers. However, the MMP3 protein, released into treated fibroblast conditioned medium, significantly increased in a dose dependent manner (Fig 3B). Figure 3C depicts the quantitative analysis of the data shown in figures 3B for MMP3 secretion. As it is shown in this figure, again KynA has the highest stimulatory effect on the MMP3 secretion as compared to other tryptophan metabolites examined and D-Kyn fails to stimulate MMP3 secretion by fibroblasts.

**Figure 4:**
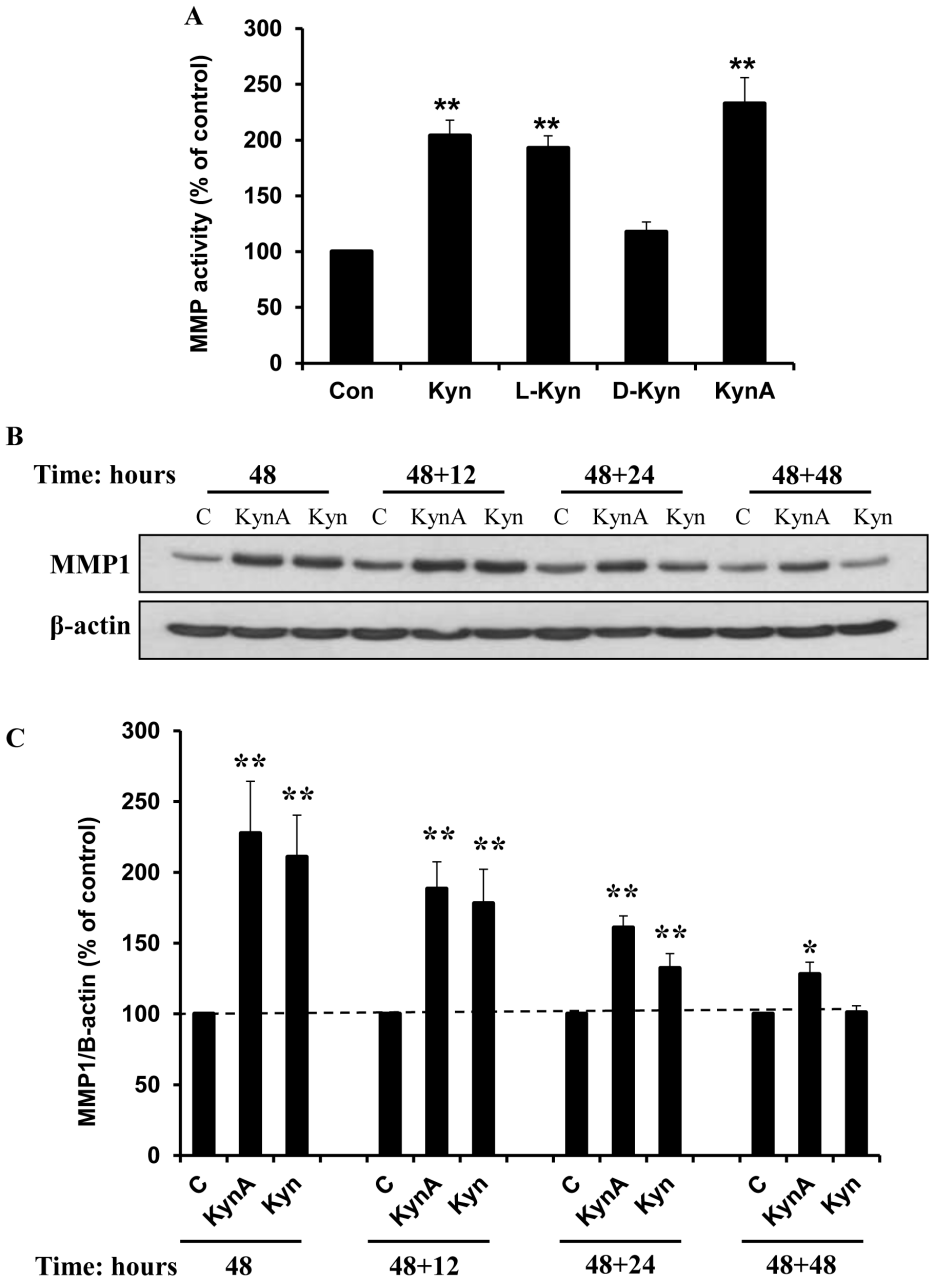
Stimulatory effect of Kynurenines on MMP activity and Kynurenines lasting effect on MMP1 expression. **A**: The effect of kynurenines of MMP activity. To determine MMP activity in the fibroblasts conditioned medium, cells were treated with 100 μg/ml of KynA, Kyn, L-Kyn or D-Kyn for 48 hours and MMP activity was evaluated using SensoLyte Plus 520 generic MMP Assay Kit (** P-value<0.001, n = 4). **B**: Kynurenines lasting effect on the MMP1 expression. To determine the lasting effect of kynurenines on MMP1 expression, fibroblasts were treated with KynA or Kyn (100 μg/ml) for 48 hours. The medium was replaced and cells were harvested immediately, 12, 24, and 48 hours after treatment removal. The MMP1 expression in dermal fibroblasts was evaluated using western blotting. **C**: MMP1/β-actin expression ratio was calculated in treated fibroblasts. Data is mean±SEM of 4 independent experiments (* P-value<0.05 and ** P-value<0.01, n = 4).

Since the enzymatic activity of MMPs is of primary importance when investigating an antifibrogenic drug, a protease activity assay was performed using treated fibroblast conditioned medium. Fibroblasts were first treated with different kynurenines (100 μg/ml) for 48 hours prior to being subjected to the SensoLyte Plus 520 generic MMP Assay kit. Consistent with previous data generated by Western blot analysis, fibroblast treatment with Kyn, L-Kyn or KynA significantly increased the MMP activity in comparison to cells treated with either D-Kyn or untreated cells (Fig. 4A).

Considering the comparable effects of KynA, Kyn and L-Kyn on ECM expression, KynA and Kyn were selected for further studies. To determine the lasting effect of KynA and Kyn on MMP1 expression in fibroblasts, these cells were treated with 100 μg/ml of the drug. Following 48 hours of treatment, the medium was changed and cells were harvested at 0, 12, 24 or 48 hours post treatment removal. The result showed a marked increase in MMP1 expression by fibroblasts in response to either KynA or Kyn treatment at 48 hours after treatment. Following the removal of Kyn and KynA, the MMP1 expression remained significantly higher than the untreated cells for another 24 hours (Fig. 4B). Interestingly, while the MMP1 protein expression gradually reduced to normal levels within 48 hours after Kyn removal, the MMP1 expression in response to KynA remained higher than controls (figure 4B). Figure 4C represents the quantitative analysis of data in figure 4B. This finding shows that KynA has a longer lasting effect on expression of MMP-1 relative to Kyn in treated fibroblasts.

**Figure 5:**
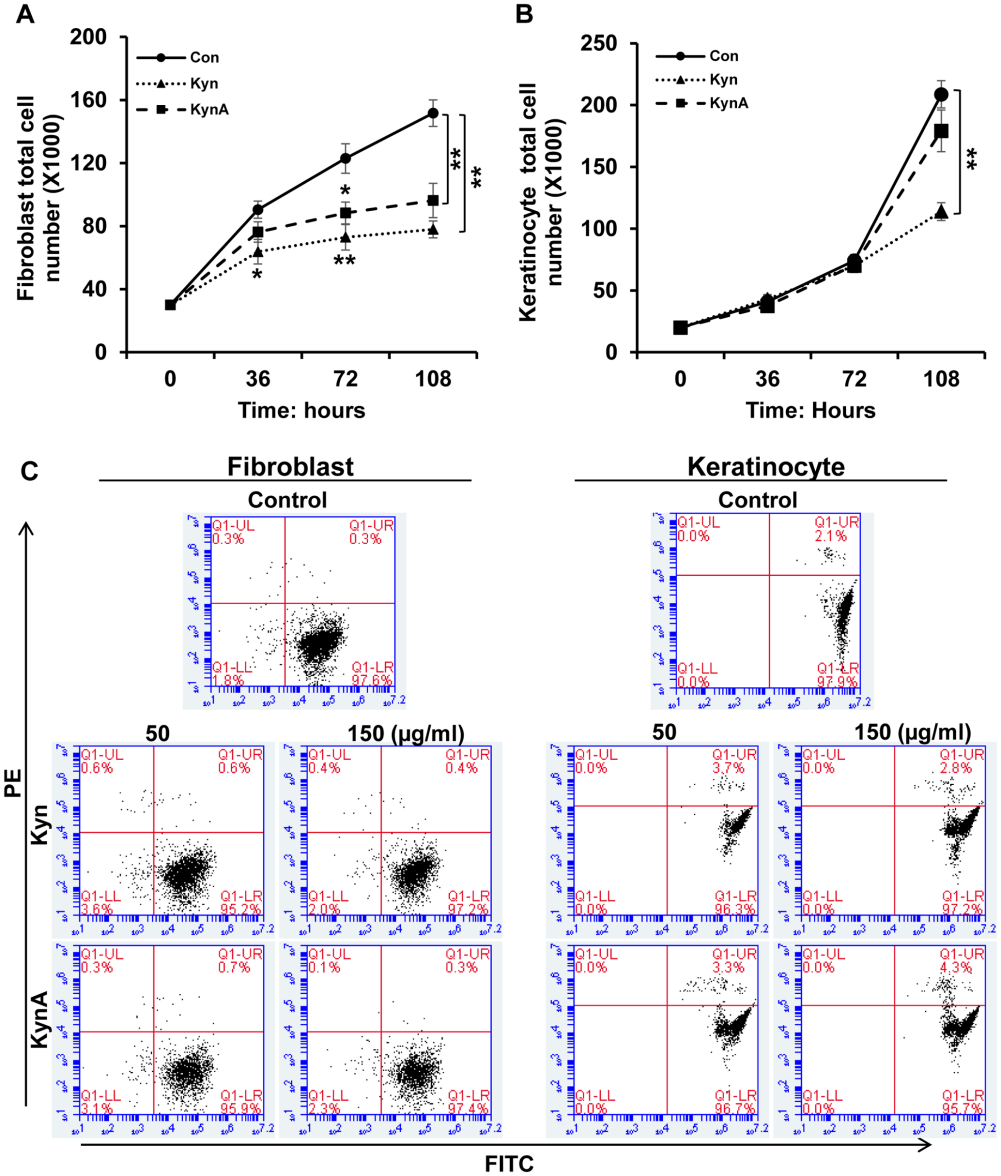
Effect of kynurenines on fibroblast and keratinocyte proliferation rate and viability. **A & B**: To determine the effect of different kynurenines on dermal cell proliferation rate, fibroblast and keratinocytes were treated with KynA or Kyn (100 μg/ml). Cells were harvested and total cell number was counted after 36, 72 and 108 hours of incubation (* P-value<0.05 and ** P-value<0.01, n = 6). **C**: Determination of cellular viability by FACs analysis using live/dead, viability/cytotoxicity assay kit. Fibroblasts and keratinocytes were either cultured in DMEM+2% FBS or DMEM+2% FBS supplemented with increasing concentrations of KynA or Kyn (50 and 150 μg/ml). The viability of cells was determined by FACS analysis following 3 days of incubation.

To evaluate the effect of Kyn and KynA on dermal cell proliferation rate, cells were treated with Kyn and KynA (100 μg/ml) and the total cell number was counted after 36, 72 and 108 hours of incubation. As shown in figure 5A, Kyn and KynA treated fibroblasts demonstrate a significant reduction in the total cell number as compared with the cells cultured in DMEM+2% FBS at all tested time points. As it is shown in figure 5B while the total number of the cells is comparable between KynA-treated keratinocytes (179.17±16.84) and the control (208.68±10.98), Kyn significantly reduce the total number of the keratinocytes (113.88±7.19) at 108 hours post treatment. The effect of increasing concentrations of Kyn and KynA on dermal cell survival was determined by flow cytometry using a viability cytotoxicity assay in which live and dead cells are stained green and red respectively. Fibroblasts and keratinocytes exposure to increasing concentrations (50 and 150 μg/ml) of kynurenines did not significantly increase the number of dead cells. This finding indicates that Kyn and KynA treatment does not compromise the viability of either keratinocytes or fibroblasts at the tested concentrations (Fig 5C).

**Figure 6:**
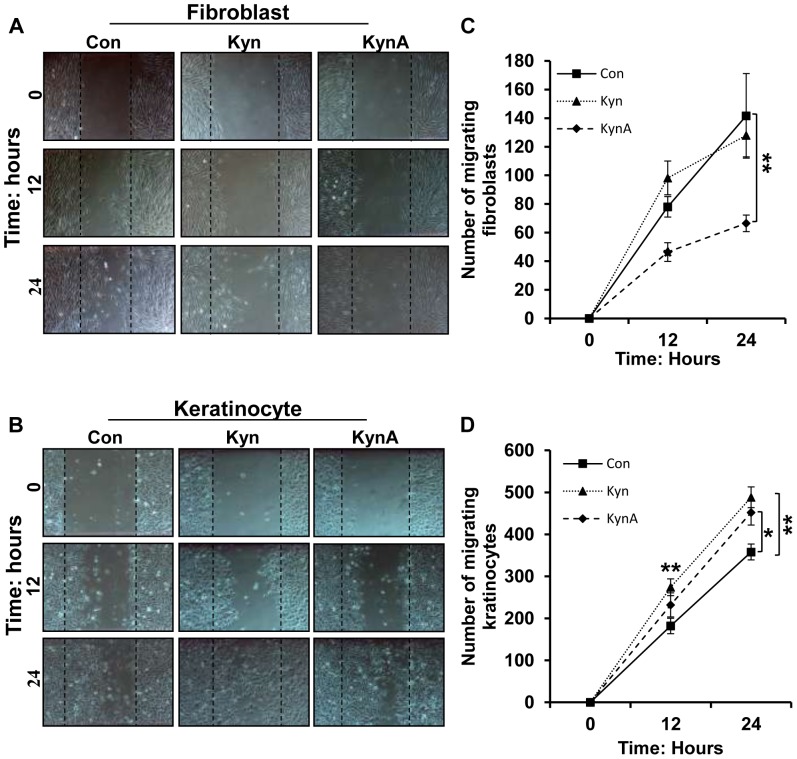
Effect of kynurenines on fibroblast and keratinocyte migration. **A & B**: Images of human fibroblasts and keratinocytes taken immediately, 12 and 24 h after addition of KynA or Kyn (100 μg/ml) in an *in vitro* wound scratch assay, respectively. **C**: Reduction of fibroblast migration in the presence of KynA after 12 and 24 hours of treatment. **D**: Enhancement of keratinocyte migration in the presence of KynA and Kyn. Cell migration was evaluated by manually counting the total number of cells migrated from the edges of the wound into the denuded area (* P-value<0.05 and ** P-value<0.01, n = 4).

To examine the effect of Kyn and KynA on dermal cell migration, wound healing scratch assay was done on fibroblasts and keratinocytes treated with Kyn or KynA (100 μg/ml). Images of cells were taken at 0, 12, and 24 hours post-treatment and the total number of the cells migrated into the open scratch area was compared between the treated and untreated cells (Fig. 6A and 6B). As it is shown in figure 6C while Kyn-treated fibroblasts demonstrate comparable migration to the control, KynA significantly reduce the number of migrating fibroblasts into the wound area compare to the control at 12 (46.4±14.58 vs. 78±17.14) and 24 hours (66.5±11.67 vs. 116±16.79) post treatment. Interestingly, both Kyn and KynA significantly increased (488±65.87 and 452±73.52, respectively) the number of migratory keratinocytes into the wound area compared to the controls (358±50.13) at 24 hours after treatment (Fig. 6D).

**Figure 7:**
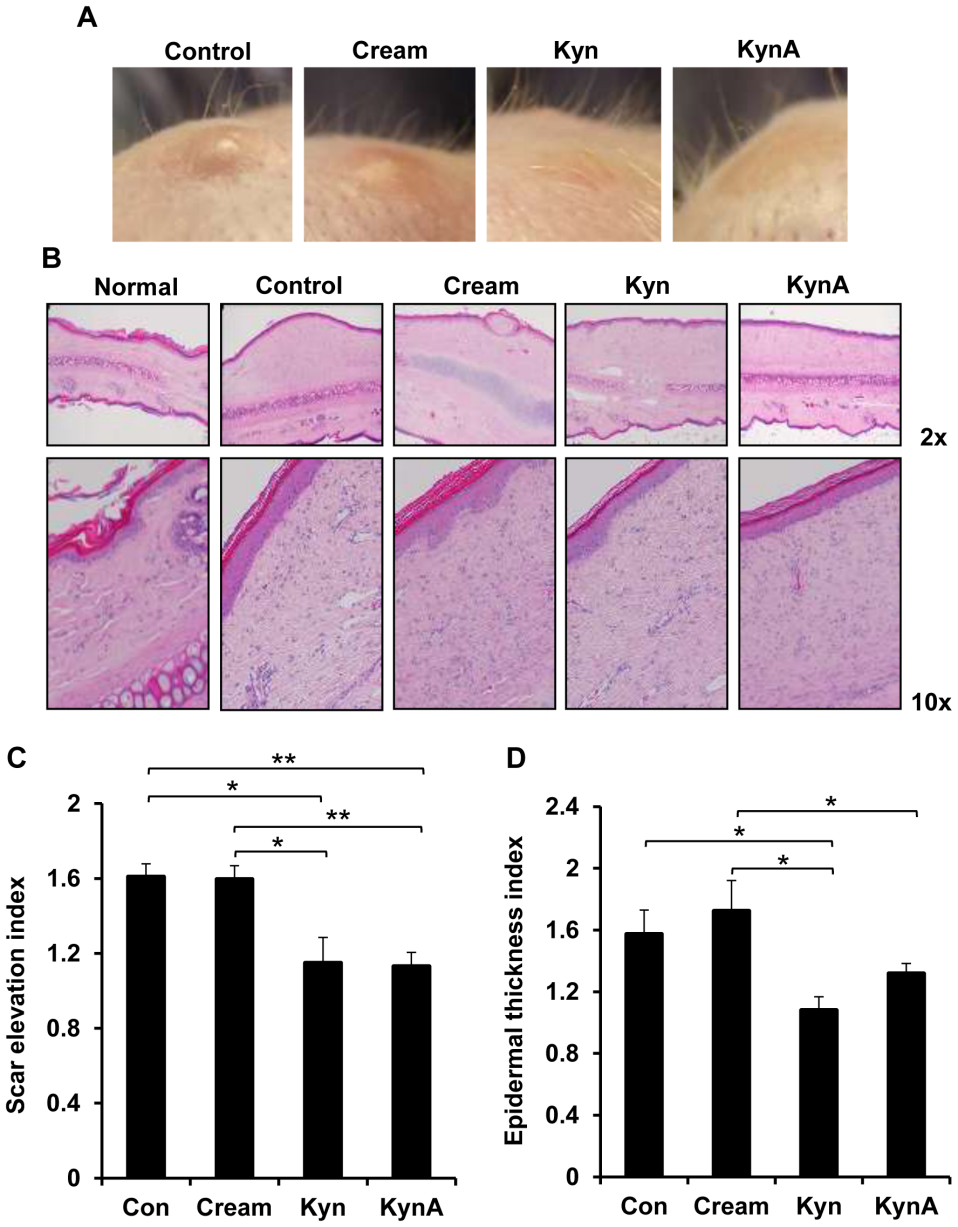
Clinical appearance and histological evaluation of wounds in rabbit ear model. A: The clinical appearance of wounds that either received nothing (Control), cream only (cream) or cream containing Kyn or KynA on day 35 post wounding. B: Tissue samples were subjected to H&E staining to determine the dermal and epidermal hypertrophy. Scar elevation index (C) and epidermal thickness index (D) was evaluated quantitatively. Uninjured rabbit ear skin was used as the normal sample (* P-value<0.05 and ** P-value<0.01, n = 4).

To validate the physiological antifibrogenic effects of topically applied KynA and Kyn, creams containing the compounds were applied on a fibrotic rabbit ear model. Four full thickness wounds were generated per ear and 3 days later wounds received daily application of either cream only or cream containing KynA or Kyn (500 μg/ml). Wounds in the control group remained untreated and healed by secondary intention. Clinical and histological examination of wounds demonstrated a significant decrease in scar elevation in Kyn or KynA treated wounds compared with those of control and cream treated wounds (Fig. 7A). As it is shown in figure 7B, dermal and epidermal layers are markedly thinner in wounds receiving topical application of either Kyn or KynA compared to the untreated wounds and wounds treated with cream only. H&E stained tissue sections were examined to determine the SEI and ETI. The SEI was decreased significantly in wounds treated with topical Kyn (SEI of 1.15±0.24) or KynA (SEI of 1.13±0.13) in comparison to the wounds treated with cream only (SEI of 1.6±0.13) or untreated control wounds (SEI of 1.61±0.12). The SEI decrease corresponds to a reduction in scar hypertrophy of 28.56% and 29.75% in Kyn and KynA treated wounds respectively (Fig. 7C). Epidermis is significantly thicker in untreated wounds (ETI of 1.57±0.16) or wounds treated with cream only (ETI of 1.72±0.2) in comparison to uninjured skin. Epidermal hypertrophy decreased markedly in wounds that received Kyn (ETI of 1.08±0.09) compared to the wounds treated with cream only or untreated control wounds (Fig. 7D). Topical KynA slightly reduced the ETI compared to the control untreated wounds, but this reduction did not reach the statistical significance (ETI of 1.32±0.07). These scars exhibited a reduction in ETI of 31.30% or 16.19% when treated with Kyn or KynA cream respectively.

**Figure 8:**
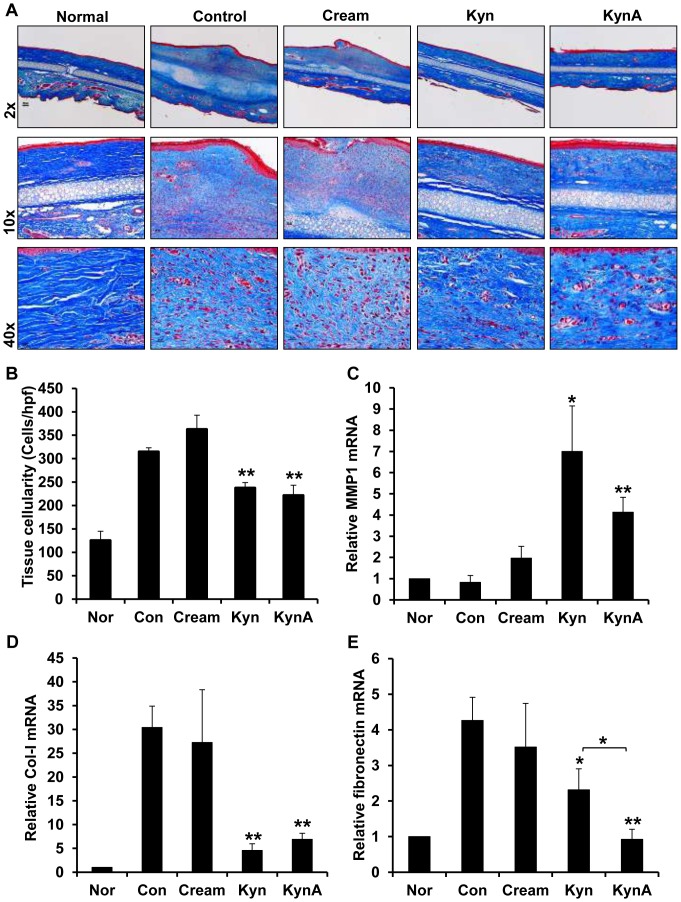
Effect of Kyn and KynA topical application on collagen deposition, tissue cellularity and ECM expression. A: Evaluation of collagen deposition in tissue samples using Masson's Trichrome staining at day 35 post-wounding. In this staining collagen fibers are stained blue, keratin and muscle fibers are stained red, and cell cytoplasm and nuclei are stained light pink and dark brown, respectively. B: Quantification and statistical analysis of tissue cellularity. Q-PCR analysis of relative MMP1 (C), type-I collagen (D) and fibronectin (E) mRNA expression in tissue samples (* P-value<0.05 and ** P-value<0.01, n = 4).

Collagen deposition was also evaluated using Masson's Trichrome-staining in which collagen is stained blue and cell nuclei are stained dark brown (Fig. 8A). The effect of topical KynA and Kyn on dermal cellularity was evaluated by counting the number of nuclei per 20 of the high-power field from each group of wounds (Fig. 8B). The total cell count was the highest in untreated wounds and wounds treated with cream only with an average of 314.56±8.66 and 362.34±30.4 cells/hpf, respectively. Dermal cellularity was markedly reduced in wounds treated with Kyn or KynA, 237.38±11.6 and 221.38±21.7 cells/hpf respectively and corresponds to a respective 24.5% and 29.6% reduction in dermal cellularity (Fig 8B). Modulation of ECM expression in response to topical Kyn and KynA application was evaluated using Q-PCR. The result demonstrated that wound treatment with either KynA or Kyn leads to a significant increase in the expression of MMP1 and decrease in the type-I collagen mRNA expression in comparison with untreated wounds or wounds treated with cream only (Fig. 8C and D, respectively). In general the Kyn and KynA have a comparable effect on type-I collagen and collagenase (MMP1) expression *in vivo*. Topical application of KynA and Kyn markedly reduce fibronectin mRNA compared to wounds treated with cream only or control wounds (Fig. 8E). Also as it is shown in this figure, KynA is more effective in suppressing the fibronectin mRNA in comparison to Kyn (78% vs. 45% in fibronectin mRNA reduction relative to control wounds).

## Discussion

Fibrosis is a pathological scarring process associated with exaggerated ECM production, abnormalities in ECM degradation and high tissue cellularity. Ideally, antifibrotic strategies aim to reduce the ECM accumulation via suppressing ECM biosynthesis or promoting matrix degradation. Our previous study regarding the antifibrotic effects of local IDO expression [11] led to identification of Kyn as an antiscarring agent [12]. Topical application of Kyn as an antifibrotic therapy is very attractive; however, the possible adverse effects of Kyn administration [13], [17], [18] prompted us to investigate comparable antifibrogenic compounds. KynA, the end product of Kyn's deamination via Kyn aminotransferase, was our candidate of choice considering its neuroprotective effects and relatively low toxicity. Moreover, the enantiomers L and D-Kyn were also investigated in order to identify the stereospecificity of the drug. We compared the biological activity of KynA and different Kyn enantiomers in terms of: 1) induction of ECM production and degradation, 2) the lasting effect of Kyn and KynA on MMP1 expression in dermal fibroblasts, and 3) on the rate of dermal cell proliferation, viability and migration. Moreover, we compared the antifibrotic effect of KynA to Kyn in a fibrotic rabbit ear model.

Excessive collagen accumulation is one of the main features of HSCs. Local modulation of ECM expression has shown a promising impact on the treatment of fibrotic diseases [6], [11], [27]. Previous studies by our group [3], [4] demonstrated higher levels of collagen type-I and collagen type-III expression in HSC samples. MMP1, 8 and 13 are the only enzymes able to initiate the degradation of interstitial collagens, type I, II and III [28]. Among these enzymes MMP1 is the only collagenase abundantly expressed by human fibroblast [29] during wound healing to remodel the ECM. In this study we showed that the tested kynurenines, with the exception of D-Kyn, not only suppress type-I collagen and fibronectin expression but also increases the rate of ECM degradation via increasing the expression and activity of MMP1 (collagenase).

In addition to MMP1, scar formation is highly dependent upon lack of MMP3 expression. Unlike MMP1, MMP3 has a wide array of ECM substrates such as fibronectin, proteoglycan, laminin, type IV, IX and X collagen [28], [30]. MMP3 was also reported to activate other pro-metalloprotease including pro-MMP1 [31], [32]. Li *et al*. [25] demonstrated that variations in MMP3 protein expression is mainly detectable in fibroblast conditioned medium not the cell lysate. Therefore in this study fibroblast conditioned medium was used to evaluate the effect of kynurenines on MMP3 expression. Although the level of MMP3 protein was significantly increased in conditioned medium in response to kynurenines, the MMP3 mRNA expression was not significantly affected by kynurenines. This finding suggests that kynurenines modulate MMP3 expression at the post-translational level.

Stereospecificity is fundamental to many biological reactions. A mounting body of evidence shows that chiral enantiomers differ significantly in biological activity, pharmacodynamics, pharmacokinetics, and toxicity. Comparison of different Kyn isomers effect on ECM expression revealed that L-Kyn is the active enantiomer of the drug. In contrary to our expectation to observe higher ECM modulation in response to L-Kyn, fibroblasts treated with the same dose of L-Kyn and Kyn, which is a racemic 1∶1 mixture of two enantiomers, demonstrated comparable ECM expression. It has previously been reported that in some cases the replacement of the racemic drug with the active enantiomer does not lead to the expected increase of drug potency [33], [34]. This might be explained by direct pharmacodynamic or pharmacokinetic competition/interaction between two enantiomers. For instance, one of the isomers can provide specific protective effects for the potent enantiomer and facilitate its physiological effects. Further pharmacological studies are required to better understand the interaction between Kyn enantiomers. The presented data also revealed that KynA has comparable effects to Kyn *in vitro* regarding the modulation of ECM expression. Although Kyn and KynA have different molecular structures, they both bind to aryl hydrocarbon receptor (AhR), a ligand-activated transcription factor, which might explain their comparable effects on fibroblasts [35], [36]. Several studies revealed the pivotal role of AhR signalling in ECM metabolism including stimulation of MMPs (MMP1, MMP9 and MMP13) expression and suppression of collagen production [37], [38], [39], [40]. A study by Anguilera-Montilla [41] revealed the contribution of AhR in the MEK/ERK signalling pathway. Also we have recently shown that Kyn induced MMPs expression is dependent on MEK-ERK1/2 MAPK signalling pathway activation [12].

Direct comparison of the lasting effects of a single dose of either Kyn or KynA demonstrated that KynA administration outlasts Kyn in its antifibrogenic response. Therefore, less frequent application of KynA is required to get the same effect compared to Kyn which can reduce the possible adverse effects of the medication. The added efficacious duration of KynA suggests a longer therapeutic half life for KynA, which may translate to a more feasible dosing regime in the clinical setting.

*In vitro* wound healing scratch assay showed enhanced keratinocyte migration in response to Kyn and KynA treatment. The enhanced keratinocyte migration may lead to accelerated wound re-epithelialization, reduced wound contraction and scar formation [42], [43].

In the current study we found that KynA and Kyn with concentrations over 25 μg/ml significantly increase the expression of MMPs and suppress the type-I collagen and fibronectin synthesis *in vitro*. Therefore, in order to test the antifibrotic effects of KynA and Kyn in a rabbit ear fibrotic model, wounds were daily treated with 50 μg of either Kyn or KynA/100 μl of cream per wound. Since kynurenines increase the expression of MMPs and suppress the expression of type-I collagen, application of the cream right after wounding may have compromised the formation of granulation tissue and impeded the early phases of wound healing. On the other hand, application of kynurenines at the latter stages of wound healing might not be highly effective in prevention of scar formation. Therefore, in this study topical Kyn and KynA application started at day 3 post wounding in order to allocate enough time for granulation tissue formation and at the same time target the inflammation and proliferation phase of wound healing. Results from clinical and histological studies demonstrated that treated wounds are fully epithelialized and healed by day 35 post wounding. As such Kyn or KynA treated wounds did not show any significant delay in wound closure indicating that the application of kynurenines at the mid-stages of wound healing does not delay the healing process. Topical Kyn and KynA application on the rabbit ear significantly improved the wound healing outcome through increasing the expression of MMP1 and suppressing the expression of type-I collagen and fibronectin. Reduced collagen deposition was confirmed by down-regulation of type-I collagen and up-regulation of MMP1 expression in KynA or Kyn treated wounds. Results from our *in vivo* experiments were further supported by our previous studies [11], [44], [45] showing that wound treatment with IDO-expressing skin substitute not only accelerates the wound healing process, as a skin substitute, but also decreases the inflammation and scar formation. In addition to evaluating the effect of KynA treatment on collagenase and collagen expression it was found that KynA and Kyn suppress the expression of fibronectin *in vitro* and *in vivo*, and further, that KynA is more effective in reducing the fibronectin mRNA. Fibronectin is an abundant component of the provisional matrix regulates different aspects of wound healing including cell attachment, migration and differentiation, matrix organization and wound contraction [46]. Exaggerated fibronectin expression and accumulation are pathological features of fibrotic disorders [47], [48], [49], [50]. By acting as a potent chemoattractant [51], fibronectin recruits fibroblasts into the wound bed and induces their differentiation into myofibroblasts [52]. Also it has been shown that fibronectin promotes epithelial mesenchymal transition (EMT), a very important mechanism in fibrosis [53], [54]. Inhibition of fibronectin accumulation has been used as an effective strategy to prevent renal fibrosis *in vivo* [55].

Another hallmark of fibrosis is high cellularity. Exaggerated fibroblast proliferation and abnormalities in myofibroblasts apoptosis can lead to excessive cellularity at the wound bed and scar formation [56]. Several studies revealed that application of antifibrogenic agents [57], [58] reduces fibroblast proliferation and reduces overall wound cellularity thereby improving wound healing outcome. Our *in vitro* studies revealed Kyn and KynA effectively reduce the proliferation rate of the primary fibroblasts, without compromising cellular viability. The inhibitory effect of kynurenines on fibroblast proliferation was further confirmed by a significant reduction in tissue cellularity in response to KynA or Kyn treatment. *In vitro* wound healing assay also showed a significant reduction in fibroblast migration in response to KynA treatment which may play a key role in tissue cellularity reduction in KynA treated wounds.

In conclusion, the results of this study demonstrate the antifibrogenic efficacy of KynA, Kyn and L-Kyn both *in vitro* and *in vivo*. For the first time, the stereospecific antifibrogenic effect of Kyn was also observed through independent assays of the L/D enantiomers demonstrating that D-Kyn has no therapeutic effect. KynA and Kyn treatments enhance ECM remodeling via increasing the expression of ECM degrading enzymes (MMP1 and MMP3) and suppression of type-I collagen and fibronectin production. It was further concluded that Kyn and KynA administration in a topical cream at the mid-stage of wound healing offer a therapeutic strategy to reduce scarring *in vivo*, reducing SEI and ETI of healed wounds. Considering the possible adverse effects of Kyn, together with the prolonged therapeutic effects of a single KynA dose and the enhanced suppression of fibronectin, KynA has demonstrated, in this study, to be a suitable antifibrogenic candidate drug to improve healing outcome in patients that suffer from hypertrophic scarring and keloids.

## Supporting Information

Figure S1 **Reduction of soluble collagen level in KynA and Kyn treated fibroblast conditioned medium.** To determine the effect of Kynurenines on soluble collagen production by fibroblasts, cells were treated with KynA and Kyn (50 and 150 μg/ml). Following 96 hours of incubation the amount of collagen in the cell culture medium was measured using Sirius Red collagen detection kit. Results are expresses as the amount (μg) of soluble collagen per 10^5^ cells (*P-value<0.05 and **P-value<0.01, n = 4).
(TIF)
