# Supplementary figures and images for: Anti-Scarring Properties of Different Tryptophan Derivatives
Source: PLoS One. 2014 Mar 17;9(3):e91955. doi: 10.1371/journal.pone.0091955 (PMC3956813; doi:10.1371/journal.pone.0091955)

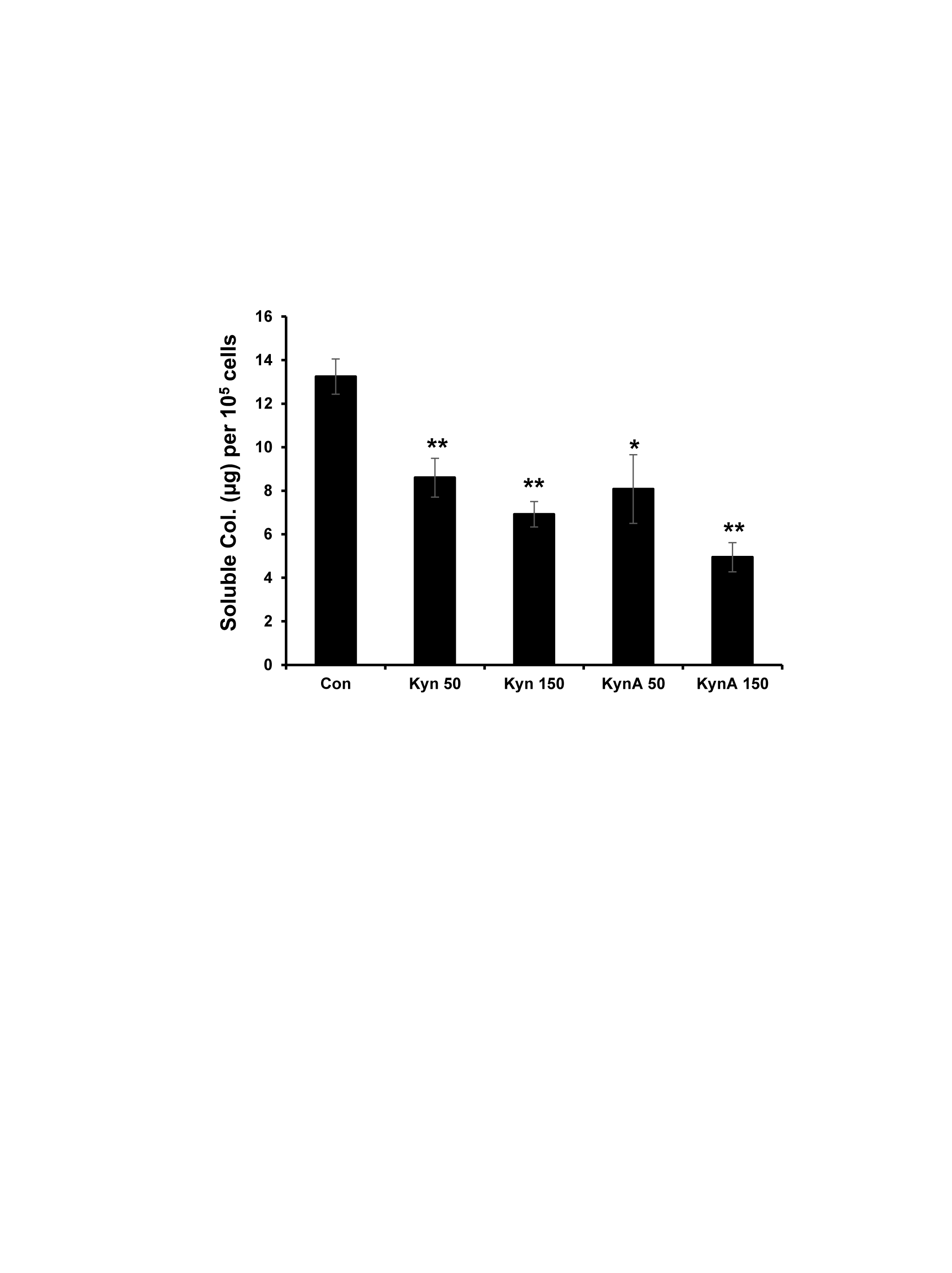

Supplement: Figure S1 — Reduction of soluble collagen level in KynA and Kyn treated fibroblast conditioned medium. To determine the effect of Kynurenines on soluble collagen production by fibroblasts, cells were treated with KynA and Kyn (50 and 150 μg/ml). Following 96 hours of incubation the amount of collagen in the cell culture medium was measured using Sirius Red collagen detection kit. Results are expresses as the amount (μg) of soluble collagen per 105 cells (*P-value<0.05 and **P-value<0.01, n = 4). (TIF) [file pone.0091955.s001.tif]
